# Key role for ubiquitin protein modification in TGFβ signal transduction

**DOI:** 10.3109/03009734.2012.654858

**Published:** 2012-04-19

**Authors:** Miriam De Boeck, Peter Ten Dijke

**Affiliations:** ^1^Department of Molecular Cell Biology and Centre for Biomedical Genetics, Leiden University Medical Center, Postbus 9600, 2300 RC, Leiden, The Netherlands; ^2^Uppsala University and Ludwig Institute for Cancer Research, Box 595, 75124, Uppsala, Sweden

**Keywords:** BMP, DUB, E3 ligase, proteasome, signaling, Smad, Smurf, TGFβ, TRAF6, ubiquitin

## Abstract

The transforming growth factor β (TGFβ) superfamily of signal transduction molecules plays crucial roles in the regulation of cell behavior. TGFβ regulates gene transcription through Smad proteins and signals via non-Smad pathways. The TGFβ pathway is strictly regulated, and perturbations lead to tumorigenesis. Several pathway components are known to be targeted for proteasomal degradation via ubiquitination by E3 ligases. Smurfs are well known negative regulators of TGFβ, which function as E3 ligases recruited by adaptors such as I-Smads. TGFβ signaling can also be enhanced by E3 ligases, such as Arkadia, that target repressors for degradation. It is becoming clear that E3 ligases often target multiple pathways, thereby acting as mediators of signaling cross-talk. Regulation via ubiquitination involves a complex network of E3 ligases, adaptor proteins, and deubiquitinating enzymes (DUBs), the last-mentioned acting by removing ubiquitin from its targets. Interestingly, also non-degradative ubiquitin modifications are known to play important roles in TGFβ signaling. Ubiquitin modifications thus play a key role in TGFβ signal transduction, and in this review we provide an overview of known players, focusing on recent advances.

## Introduction

One of the major regulators of cell communication in all multicellular organisms is transforming growth factor β (TGFβ). TGFβ is the prototypic family member of 33 secreted, structurally related human cytokines. They are involved in the regulation of cell proliferation, differentiation, apoptosis, and motility of diverse cell types. Whereas practically all human cell types respond to TGFβ, the actions of certain family members are more cell type-selective. The importance of proper TGFβ signaling is highlighted by the observation that perturbed TGFβ signaling results in tumorigenesis, and many different mutations or other alterations in TGFβ signaling components have been identified in human cancers. Intriguingly, TGFβ plays a dual role in cancer development and progression. During the early stages of tumorigenesis, TGFβ acts as a tumor-suppressor by inhibiting proliferation. Later in cancer progression, TGFβ has pro-angiogenic and immunosuppressive effects, and it promotes metastasis by inducing epithelial to mesenchymal transition (EMT). Cancer cells that manage to evade the anti-proliferative effects of TGFβ and simultaneously maintain the tumor-promoting effects benefit from this distorted signaling. The wide variation in cellular responses to TGFβ demonstrates the complexity of the intracellular signaling pathways. By studying TGFβ signaling and its cross-talk to other pathways, we gain insight into the regulation of cell behavior and consequently in the mechanisms underlying cancer development. In this review we will focus on the regulation of TGFβ signaling by the ubiquitin system. We will discuss several mechanisms by which TGFβ pathway components are targeted for degradation as a way of negative regulation. Yet TGFβ signaling can also be enhanced via the degradation of negative regulators, and ubiquitination even plays a crucial part downstream of TGFβ in various cross-talk pathways. Furthermore, the roles of deubiquitination and non-degradative ubiquitination will be discussed. Finally, we will touch upon the opportunities these regulatory mechanisms give us for pharmacological intervention.

## Transforming growth factor β signaling

**Figure 1.**
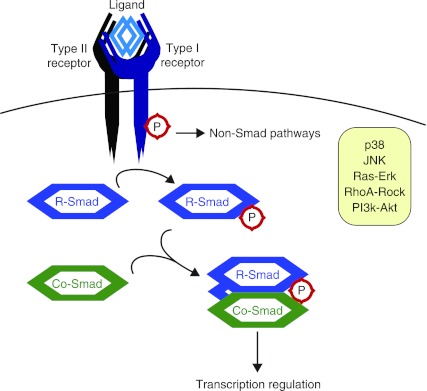
The transforming growth factor β (TGFβ) pathway. TGFβ ligands function as dimers and signal through type I and type II serine/threonine kinase receptors. Upon ligand binding, the receptors form heterotetrameric complexes allowing the type II receptors to phosphorylate and activate the type I receptors. Subsequently, receptor-activated Smads (R-Smads) are recruited to the type I receptors and phosphorylated. The activated R-Smads associate with the Co-Smad, Smad4, and this heteromeric complex translocates to the nucleus to participate in the transcriptional control of specific target genes (94). TGFβ can also activate various non-Smad signaling pathways.

The TGFβ family of cytokines consists of many members including bone morphogenetic proteins (BMPs), growth and differentiation factors (GDFs), and activins. TGFβ ligands signal through receptors and Smads and regulate target gene transcription (Figure 1). The differential expression of co-activators and co-repressors in the various cell types gives rise to the wide range in cellular responses. Besides regulating gene transcription through Smad signaling, TGFβ can also activate various non-Smad pathways (1,2). Some of these pathways operate independently of Smads; yet others co-operate or even interfere with Smad signaling. The p38 and Jun N-terminal kinase (JNK) mitogen-activated protein (MAP) kinase pathway is activated upon TGFβ stimulation via the ubiquitin ligase tumor necrosis factor (TNF) receptor-associated factor 6 (TRAF6) and TGFβ activated kinase 1 (TAK1). TGFβ can also activate the Ras-Erk-MAPK pathway, depending on cellular context. This pathway stimulates TGFβ-induced EMT, yet it competes with Smad-dependent signaling in regulating cell proliferation. Other pathways affected by TGFβ include RhoA-Rock and the phosphoinositide 3-kinase (PI3K)-Akt pathway.

Since TGFβ signaling is important for a wide variety of cell functions, it must itself be tightly regulated. It cannot simply be regarded as an on/off switch, because both signal strength and duration are important factors affecting the cellular response outcome. The cellular response to TGFβ is highly dependent on the expression levels of receptors (3), Smads, transcription factors, and other signal regulators. Phosphorylation is an important modification in signaling pathways; therefore dephosphorylation is involved in regulating signal transduction as well. And finally, as the abundance of specific proteins and the half-life of activated signaling molecules are crucial for determining the response, targeted protein degradation is indispensable for regulating cell sensitivity and signal duration. A major pathway to achieve this is the ubiquitin-proteasome system.

## The ubiquitin system

**Figure 2.**
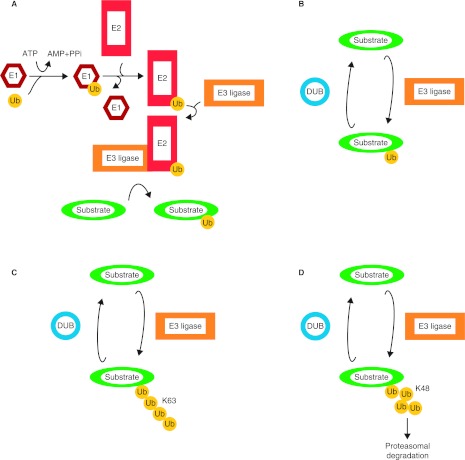
The ubiquitin system. A: Free ubiquitin is bound by the active cysteine residue of an E1 ubiquitin-activating enzyme, and this process requires ATP. Next, Ub is transferred onto the active cysteine of an E2 ubiquitin-conjugating enzyme. Finally, Ub is transferred onto a lysine residue of the target protein by an E3 ubiquitin ligase. Ubiquitin can be conjugated as mono-ubiquitin (B) or in chains such as K63 chains (C) or K48 chains (D), the last-mentioned known for targeting substrates for proteasomal degradation.

Ubiquitination is a post-translational modification of proteins, which can affect their stability, activity, and cellular localization. Ubiquitin (Ub) is a small, 8.5 kDa, protein that is conjugated onto target proteins via its C-terminal glycine (Gly76) residue. Ub is produced in a precursor state of linear chains of Ub moieties or fused to ribosomal proteins. The activity of deubiquitinating enzymes (DUBs) is required to produce free Ub, either by processing precursor Ub or by recycling Ub by removing it from its targets (4). The activity of E1 activating enzymes, E2 conjugating enzymes, and E3 ubiquitin ligases is necessary to conjugate ubiquitin onto its substrates (5). Functional differences between the many different E2 and E3 enzymes lead to substrate and chain specificity. Ub can be conjugated as a single moiety (mono-ubiquitination), or it can form chains, using all internal lysines (K), including K11, K48, and K63 (Figure 2), or a head-to-tail linear chain. All ubiquitin chains, with the exception of K63 chains, have been described to target proteins for proteasomal degradation, thereby regulating their stability. Furthermore, linkage-specific ubiquitin chains have been shown to affect protein–protein interactions and can thereby regulate protein function (6).

E3 ligases display both substrate and chain specificity. While one E3 ligase may preferentially target its substrates for degradation via K48 Ub chains, another may regulate the localization of its targets via mono-ubiquitination. Directly opposing the conjugating function of E3 ligases are the deubiquitinating enzymes (DUBs). DUBs are proteases that remove Ub moieties from their targets. In case of the K48 Ub chain-mediated proteasomal degradation pathway, DUBs remove the Ub chain and stabilize the protein. DUBs also show substrate and chain specificity and therefore represent another layer of regulation of the ubiquitin system.

## Negative regulation of TGFβ signaling by the ubiquitin-proteasome system

TGFβ induces the expression of various genes, among which are negative regulators, such as I-Smads (7,8) and Smurfs that function in a feedback mechanism. Smurfs are HECT (homologous to the E6-accessory protein C-terminus)-type E3 ligases that are known regulators of the TGFβ pathway. E3 ligases regulate their own abundance via autoubiquitination. Under steady-state conditions, Smurf2 inhibits its own ubiquitinase activity and is thereby stabilized (9). Upon binding of Smad7, Smurf2 becomes activated. When TGFβ signaling is active, interacting Smurfs and I-Smads are exported from the nucleus to the cytoplasm. I-Smads recruit Smurfs to the active TGFβ receptor complexes, and Smurfs target the complexes for degradation (10–12). CD109 has recently been identified as a negative regulator of TGFβ signaling by enhancing receptor ubiquitination, in a ligand-dependent manner, by Smurf2 and Smad7 (13,14). The E3 ligases WWP1 (WW domain-containing protein 1) and NEDD4-2 (neural precursor cell expressed, developmentally down-regulated 4–2) have also been shown to be recruited to TGFβ receptor complexes by Smad7 (15,16). The subsequent ubiquitination and degradation of the receptors leads to an inhibition of all downstream pathways.

Smurfs also regulate the canonical TGFβ pathway at the level of Smad signaling. Smurf1 was shown to ubiquitinate Smad1 and Smad5 (11,17), while Smurf2 ubiquitinates Smad1 and Smad2 under steady-state conditions (18,19). The abundance of Smad1 and Smad3 is also regulated by the E3 Ub ligase U-box-containing carboxyl terminus of Hsc70-interacting protein (CHIP) (20,21). For Smad3, it was shown that a complex of Axin and glycogen synthase 3-β (GSK3β) affects the ubiquitination, thereby linking R-Smad levels to other cellular pathways in which Axin/GSK3β function (22). These ubiquitin-mediated degradation pathways are important for controlling the sensitivity of the cell to TGFβ by adjusting the absolute and relative abundance of different Smads before the initiation of signaling.

In the presence of TGFβ, the active receptor complexes phosphorylate R-Smads, which signal in the nucleus. To limit signaling by Smad complexes, the phosphorylation of R-Smads increases their susceptibility to ubiquitination by E3 ligases such as Smurfs. Phosphorylated Smad1 is subsequently phosphorylated by MAP kinase and GSK3β (23) to increase its ubiquitination by Smurf1. Also Smad2 becomes more susceptible to ubiquitination by Smurf2 after phosphorylation. Pin1 interacts with phosphorylated Smad2 and Smad3 and enhances their ubiquitination by Smurf2 (24). Phospho-Smad2/3 are also targeted for degradation by NEDD4L (25). Furthermore, Smad3 is targeted for degradation by ROC1-SCF^Fbw1a^, and this is dependent on TGFβ (26). In the nucleus, R-Smads are phosphorylated by CDK8 and CDK9 to enhance transcription, yet these modifications increase Smad ubiquitination (27). The subsequent phosphorylations on activated R-Smads by several kinases regulate the affinity for E3 ligases and therefore the half-life of activated Smads (28). E3 ligase Arkadia was shown to ubiquitinate phospho-Smad2/3, even though Arkadia is generally regarded as a positive regulator of TGFβ signaling (see below). Phospho-Smad2/3 are polyubiquitinated by Arkadia after the initiation of target gene transcription, and therefore this ubiquitination step may function to efficiently terminate signaling (29).

The Co-Smad, Smad4, is required for Smad-mediated transcriptional control. By regulating the availability of Smad4, the intracellular response to TGFβ and BMP can be controlled. Various E3 Ub ligases have been identified that target Smad4 for degradation. Similar to R-Smads, Smad4 can be ubiquitinated by Smurfs, WWP1, and NEDD4-2 recruited by Smad7 (30). Binding of Jab1 induces the ubiquitination and degradation of Smad4 (31). CHIP and SCF^β-TrCp1^ can also conjugate poly-ubiquitin chains onto Smad4, which leads to its degradation and an inhibition of signaling (32,33). The importance of proper regulation of the ubiquitin-mediated proteasomal degradation of Smad4 becomes clear in many human cancers where Smad4 is often lost. Mutations in Smad4 can lead to an increase in ubiquitination and therefore render the protein unstable (34,35).

As TGFβ signaling progresses, the expression of many negative regulators, such as Smurfs, is induced, and higher protein levels of these E3 ligases increase the degradation rate of the receptors and Smads, thereby terminating signaling. Yet the ubiquitin system is also an ideal mediator of cross-talk between signaling pathways. Different signals induce the expression of I-Smads and thereby inhibit TGFβ signaling, but pathways can also modulate TGFβ signaling directly by recruiting the ubiquitin system. Estrogen was shown to inhibit TGFβ signaling by promoting proteasomal degradation of Smad2/3 by Smurf1. Estrogen receptor α directly recruits Smurf to Smads, and this pathway represents direct inhibitory cross-talk mediated by ubiquitin protein modification (36).

## Positive regulation of TGFβ signaling by the ubiquitin-proteasome system

**Figure 3.**
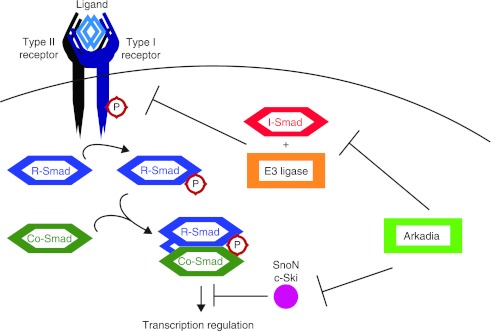
Positive regulation of TGFβ signaling by Arkadia. TGFβ signaling is inhibited by I-Smads, in co-operation with various E3 ligases, targeting several components among which the receptor complexes. SnoN and c-Ski inhibit TGFβ signaling at a later step by acting as transcriptional co-repressors. Arkadia targets I-Smad (Smad7), SnoN, and c-Ski for degradation, thereby positively regulating signaling.

An important E3 ligase for the enhancement of TGFβ signaling is Arkadia. Arkadia was identified as a positive regulator of Nodal signaling, a TGFβ family member. Arkadia targets its substrates for proteasomal degradation. Known targets for Arkadia include Smad7 (37), c-Ski, and SnoN (38), all negative regulators of TGFβ signaling (Figure 3). Smad7 was already described to recruit various E3 ligases to the TGFβ receptors and Smads, yet it also inhibits TGFβ signaling directly by inhibiting the interaction of R-Smads with the receptor complexes (7). C-Ski and SnoN inhibit Smad signaling in the nucleus by disrupting the interaction of Smads with transcriptional co-activators and by inducing inactivation of Smad complexes (39,40). They are also responsible for repressing transcription in the absence of TGFβ (41). Upon TGFβ signaling, phospho-Smads translocate into the nucleus and recruit Arkadia, but also other E3 ligases such as Smurf2 (42) and anaphase-promoting complex (APC) (43), to induce the ubiquitination of c-Ski and SnoN. The ubiquitination of c-Ski was found to be enhanced by the association of RB1-inducible coiled-coil 1 (RB1CC1) with Arkadia (44). The degradation of Smad7 by Arkadia is enhanced by Axin, a scaffold protein known for its function in Wnt signaling. Wnt negatively regulates Axin, thereby also inhibiting the ubiquitination of Smad7 and thus impacting TGFβ signaling (45). By ubiquitinating negative regulators, Arkadia stimulates TGFβ signaling.

More examples of positive regulation of TGFβ signaling via proteasomal degradation of negative regulators have been identified. TGFβ-induced factor 1 (TGIF1) is a transcriptional repressor of TGFβ signaling. Phosphorylated TGIF1 was found to be targeted for degradation by Fbxw7. Fbxw7 is the substrate recognition component of a ubiquitin ligase complex which was found to target proteins such as cyclin E, c-Myc, Notch, and c-Jun for degradation. Fbxw7 stimulates TGFβ signaling by inducing the degradation of TGIF1 (46).

Another E3 ligase involved in TGFβ regulation is WWP2. The full-length WWP2 (WWP2-FL) was shown to ubiquitinate both Smad2/3 and Smad7. Interestingly, the WWP2-N isoform stimulates the degradation of Smad2/3, yet the WWP-C isoform and WWP2-FL preferentially ubiquitinate Smad7 after TGFβ stimulation. These findings imply that depending on the specific isoforms expressed, TGFβ signaling can be either activated or inhibited (47).

## Ubiquitin as a mediator of non-Smad signaling

TGFβ exerts its effects via Smad-dependent and independent pathways. Some of the downstream functions of TGFβ signaling are dependent on the ubiquitin system. TGFβ was recently found to induce the ubiquitination and subsequent proteasomal degradation of Krüppel-like factor 4 (KLF4). KLF4 is a transcription factor involved in the regulation of core cell functions such as proliferation, differentiation, and apoptosis, and it has been implicated in carcinogenesis. TGFβ signaling induces the ubiquitination of KLF4 by Cdh1/APC, and this pathway is important for TGFβ-mediated transcription regulation (48).

Besides its function in regulating protein levels of TGFβ signaling components, Smurf1 has also been shown to function downstream of TGFβ as a regulator of RhoA signaling (49). TGFβ is known to affect the RhoA pathway, and this is important for TGFβ-induced EMT. Activated TβRII phosphorylates Par6, which then interacts with Smurf1. Smurf1 subsequently targets RhoA for degradation, and this loss of RhoA leads to hallmarks of EMT, such as the loss of tight junctions and cell polarity (50). Furthermore, Smurf1 was shown to be phosphorylated, thereby its substrate preference switched from Par6 to RhoA (51). The importance of Smurfs in the regulation of cell polarity, involving Par6 and a non-canonical Wnt pathway, is becoming increasingly clear (52,53).

Smurfs also mediate TGFβ anti-inflammatory signals together with Smad6 by targeting MyD88 for degradation (54). Smurf1 has been implicated in the regulation of inflammation due to its ability to ubiquitinate TNF receptor associated factors (TRAFs) (55,56). Smurf2 was found to associate with TRAF2 and ubiquitinate TNF receptor 2, thereby affecting downstream signaling (57). It is becoming clear that E3 ligases such as Smurfs do not just regulate a single pathway; rather they function as the effectors of various types of regulation, depending on their recruitment by other proteins. Together with I-Smads they inhibit the TGFβ pathway, yet other adaptors may recruit them to other pathways, such as RhoA and TRAFs. The substrate specificity of E3 ligases, such as Smurf1, can be regulated by post-translational modifications and cellular localization (51,58). A single E3 ligase can therefore have different functions depending on cellular context.

## Role of DUBs in TGFβ signaling

DUBs remove Ub chains or mono-ubiquitin modifications from target proteins, thereby counteracting the function of E3 ligases. They show specificity for the type of Ub modification and the substrate, yet generally they are less specific than E3 ligases. Some DUBs have been identified to target components of the TGFβ pathway. One such DUB is UCH37, which was shown to bind to Smad7 and deubiquitinate TβRI (59). It stabilizes the type I receptor and can therefore be regarded as the counterpart of E3 ligases such as Smurfs in regulating TGFβ receptor expression. UCH37 enhances early signaling and is important for TGFβ-induced migration (60). CYLD was shown to be involved in the regulation of TGFβ signaling in T cells. CYLD is a DUB that preferentially hydrolyses K63 chains and is known to inhibit JNK and NF-κB signaling. CYLD deubiquitinates Smad7 and thereby inhibits the activation of TAK1 and p38, thus inhibiting the TGFβ-induced development of regulatory T cells (61). Recently, USP15 was shown to deubiquitinate mono-ubiquitinated R-Smads (62). The mono-ubiquitination of R-Smads inhibits DNAbinding, therefore USP15 is required for proper TGFβ signaling.

Two other DUBs that have been implicated in TGFβ signal transduction are AMSH, associated molecule with the SH3 (Src homology 3) domain of SA (signal-transducing adaptor molecule) (63), and AMSH-like protein (AMSH-LP). They were shown to associate with I-Smads and inhibit their function, thereby potentiating BMP and TGFβ signaling (64,65). Furthermore, AMSH was shown to be ubiquitinated by Smurf2 via RNF11 recruitment (66). These DUBs preferentially cleave K63-linked Ub chains (67), yet this DUB activity has not been confirmed to be necessary for affecting TGFβ signaling. AMSH has been described to function in the regulation of receptor turnover by the endosomal sorting complexes required for transport (ESCRT) machinery (68). It is unclear what its targets are precisely, yet a role in receptor trafficking implies a more general function in cell signaling.

## Non-degradative ubiquitin modifications in TGFβ signaling

As previously discussed, not all forms of ubiquitination lead to proteasomal degradation of the target protein. Other types of Ub modifications, such as mono-ubiquitination or K11 and K63 chains, serve to alter the activation state of the protein, its subcellular localization, or its ability to form protein–protein interactions. In the TGFβ signaling pathway various examples of these types of post-translational modifications have been identified, which demonstrate the biological importance of this ‘alternative side’ of the ubiquitin system.

### Modulating Smad2 phosphorylation

One of the first steps in the canonical TGFβ pathway is the phosphorylation of R-Smads by the TβRI. The efficiency of this activation step was shown to be modulated via ubiquitination. Athophin 1-interacting protein 4 (AIP4) or Itch was shown to ubiquitinate Smad2 and thereby promote the phosphorylation of Smad2 by TβRI. This results in an enhancement of TGFβ signaling (69). E3 ligase Cbl-b is thought to have a similar function in T cells, since its loss reduces TGFβ-induced Smad2 phosphorylation (70,71). AIP4/Itch was also shown to bind Smad7 and recruit it to TβRI, thereby coupling Smad2 phosphorylation to signal inhibition.

### Smad4 mono-ubiquitination

**Figure 4.**
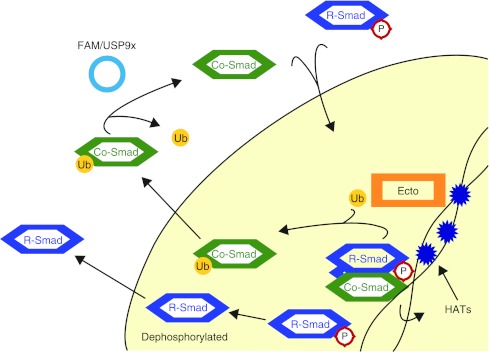
Mono-ubiquitination of Smad4. Active complexes of Co-Smad, Smad4, and phosphorylated R-Smads recruit histone acetyltransferases (HATs) to chromatin. The acetylation of histones recruits Ectodermin/TIF1γ (Ecto), which then disrupts Smad complexes and mono-ubiquitinates the Co-Smad. Released R-Smads are most likely dephosphorylated and exported to the cytoplasm. Ubiquitinated Smad4 is exported to the cytoplasm, where the deubiquitinating enzyme (DUB) FAM/USP9x removes the ubiquitin moiety. Smad4 is now ready once again to form complexes with R-Smads.

Activated Smad complexes, containing Smad4, translocate to the nucleus and bind to the promoter regions of target genes. They recruit other factors, such as histone acetyltransferases (HATs) p300/CBP to the chromatin to promote transcription. The acetylation of histones is thought to increase their affinity for proteins such as Ectodermin/TIF1γ, an E3 ligase. Bound to chromatin, Ectodermin/TIF1γ is activated to mono-ubiquitinate Smad4 at K519, and this disrupts the association of Smad4 with phospho-Smad2 (72,73). Mono-ubiquitinated Smad4 is exported to the cytoplasm (74). FAM/USP9x is a DUB that counteracts the mono-ubiquitination of Smad4. FAM/USP9x activity is required for Smad4-mediated TGFβ signaling, because it re-enables Smad4 to form complexes with R-Smads and signal in the nucleus (75). This mechanism shows a feedback loop where active Smad complexes on chromatin indirectly recruit an E3 ligase to terminate signaling (Figure 4). Moreover, the duration of Smad-chromatin binding can be regulated by the efficiency of this system. Inhibition of TGFβ signaling by Ectodermin/TIF1γ is important *in vivo* during embryonic development (72,76).

### The role of TRAF6

**Figure 5.**
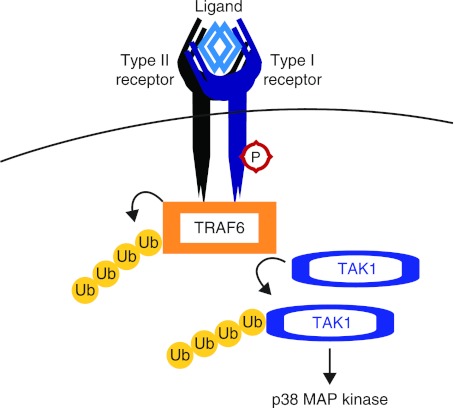
TRAF6 activates TAK1. Tumor necrosis factor receptor (TNFR)-associated factor 6 (TRAF6) binds activated TGFβ receptor complexes and is activated via K63 autoubiquitination. TRAF6 subsequently ubiquitinates and activates TGFβ-associated kinase (TAK1), which is responsible for activating non-Smad pathways such as the p38 MAP kinase pathway.

Tumor necrosis factor receptor (TNFR)-associated factor 6 (TRAF6) is a ubiquitin E3 ligase, which preferentially conjugates K63 Ub chains onto its substrates. TRAF6 interacts with TβRI and is activated via autoubiquitination, induced by TGFβ ligand binding to the receptors. Active TRAF6 ubiquitinates and thereby activates TGFβ-associated kinase (TAK1), which is important for the TGFβ-induced activation of the p38 MAP kinase pathway (Figure 5) (77). TAK1 is ubiquitinated by TRAF6 but also by TRAF2 in the TNFα pathway. Ubiquitin-specific peptidase 4 (USP4) is a DUB for TAK1 and was found to inhibit TNFα and TGFβ-induced NF-κB activation (78). This mechanism shows how the ubiquitin system can function in the cross-talk between pathways. In cancer cells, TRAF6 was also shown to ubiquitinate TβRI upon TGFβ stimulation. TβRI is subsequently cleaved by TNFα-converting enzyme (TACE), and this action creates an intracellular domain of TβRI, which functions in transcriptional complexes in the nucleus to induce the expression of EMT-related genes such as Snail and MMP2 (79). These activities of TRAF6 involve the tumor-promoting arm of TGFβ signaling.

## Concluding remarks

TGFβ is not only regulated via ubiquitination, it also relies on ubiquitination for its effect on other pathways. The ubiquitin system is a major tool for various pathways to regulate downstream mediators or other signaling pathways. E3 ligases show target specificity; yet their action *in vivo* is dependent on other proteins to act as adaptors or activators, and the specificity of E3 ligases is also dependent on the E2 enzyme providing the Ub moiety (5,49). Most E3 ligases discussed in this review have also been shown to target components of other signaling pathways.

This review focuses on ubiquitin modifications, yet other modifications such as the conjugation of small ubiquitin-like modifier (SUMO) onto TGFβ signaling components have also been found to be important. Both receptors and Smads have been shown to be SUMOylated, affecting their function (80-82). Different modifications, such as phosphorylation, acetylation, SUMOylation, and ubiquitination, can affect each other by recruiting enzymes, or they can compete with each other for binding sites. One clear example of this interplay between modifications in TGFβ signaling is the regulation of Smad7 stability. Smad7 was found to be acetylated by p300 on two lysine residues (83). These are the same residues Smurf1 uses to conjugate ubiquitin chains onto Smad7 to target it for degradation. SIRT1 is a deacetylase that counteracts the acetylation of Smad7, making the lysine residues available for ubiquitination (84). Acetylation and ubiquitination thereby compete in regulating Smad7 stability (85). In summary, the real story is longer than presented here, and with the identification of new targets, adaptors, and enzymes the overall picture is becoming increasingly complex. An overview of ubiquitin modifications discussed in this review can be found in Table I.

**Table I.** An overview of ubiquitin modifications involved in TGFβ signaling.

Target	E3 ligase/ DUB	Adaptor	Comments	References
Negative regulation				
TβRI	Smurf1	Smad6/7, FKBP12		(10,11,95)
	Smurf2	Smad7		(12)
	NEDD4-2	Smad7		(16)
	WWP1	Smad7		(15)
Smad1/Smad5	Smurf1	LMP-1, Smad6/7		(11,17,96)
Smad1	Smurf2			(19)
	CHIP			(32)
Smad2	Smurf2			(18,97)
	NEDD4-2			(16)
	WWP1	TGIF		(98)
	WWP2-FL	WWP2-N		
Smad3	CHIP			(20)
	ROC1-SCF^Fbw1a^			(26)
	WWP2-FL	WWP2-N		(47)
Smad4	Smurf1, Smurf2, NEDD4-2, WWP1	Smad2/6/7		(30)
	CHIP			(32)
	SCF^β-TrCp1^			(33)
	SCF^Skp2^		Smad4 mutants	(99)
Positive regulation				
Smad7	Smurf1/2		Affected by acetylation	(10,12,83,85)
	Arkadia	Axin		(37,45)
	WWP2-FL, WWP2-C			(47)
SnoN	Arkadia			(38,89)
	Smurf2	Smad2		(42)
	APC	Smad2/3		(43,100)
TGIF	Fbxw7			(46)
Examples of ubiquitin-mediated non-Smad signaling				
KLF4	Cdh1/APC		Induced by TGFβ	(48)
Par6	Smurf1			(51)
RhoA	Smurf1			(50-52,101)
MyD88	Smurf1/2	Smad6		(54)
TRAFs	Smurf1			(55,56)
TNFR2	Smurf2	TRAF2		(57)
DUBs in TGFβ signaling				
TβR1	UCH37			(59,60)
Smad1/2/3	USP15			(62)
Smad6	AMSH		Requirement of DUB activity not confirmed	(64)
Smad2/7	AMSH-LP		Requirement of DUB activity not confirmed	(65)
Smad7	CYLD		DUB removing K63	(61)
Non-degradative Ub modifications				
Smad2	AIP4/Itch		Increased Smad2 phosphorylation	(69)
	Cbl-b		Increased Smad2 phosphorylation. Not confirmed	(70,71)
Smad4	Ectodermin/TIF1γ	Acetylated histones	Mono-Ub disrupting complex with Smad2	(72,75,76)
	FAM/USP9x		DUB removing mono-Ub	(75)
TAK1	TRAF6		K63 activation	(77)
	USP4		DUB	(78)

As disruptions in TGFβ signal transduction are implicated in a wide variety of cancers and the ubiquitin system is important for the regulation of this pathway, it does not come as a surprise that in several cancers dysregulations of E3 ligases such as Smurfs and Arkadia have been found (86,87). In various tumors, increased Smurf expression leads to decreased Smad levels, and this affects tumor progression and correlates with poor prognosis (88). A loss of Smad4 expression is a common finding in many cancers. This loss can be caused by mutations which make it more prone to ubiquitination, thereby destabilizing Smad4 (34,35). In some tumors, TGFβ signaling is inhibited by the over-expression of a transcriptional co-repressor such as SnoN. This over-expression of SnoN was found to be caused by a loss of Arkadia and thereby a lack of Arkadia-mediated SnoN degradation. A restoration of Arkadia expression rescued TGFβ signaling in these cells (89,90). A loss of Fbxw7 and subsequent increase in TGIF1 expression has also been implicated in cancer (46). E3 ligases, and also DUBs (91), therefore represent a new class of potential oncogenes and tumor suppressors.

The ubiquitin system can be targeted pharmacologically at different levels. The only drug now being used in the clinic is bortezomib, a general proteasome inhibitor. This drug has cytotoxic effects due to the non-specific inhibition of protein degradation. It is prescribed for multiple myeloma and mantle cell lymphoma, and more proteasome inhibitors are currently under investigation as anti-cancer drugs. Yet the ubiquitin system has the potential of providing us with specific drug targets (92,93). Small molecule E3 ligase inhibitors can potentially rescue specific proteins from proteasomal degradation. An example currently being investigated is the E3 ligase MDM2, which targets the tumor suppressor p53 for degradation. But also Smurfs are interesting targets, as they are found to be over-expressed in certain cancers. DUBs are proteases and are therefore more easily targeted specifically by inhibitors. Some DUB inhibitors, such as inhibitors of USP7, are being investigated as anti-cancer drugs. Small molecule inhibitors targeting E3 ligases or DUBs involved in the regulation of TGFβ signaling could prove useful in counteracting perturbations in TGFβ signaling commonly found in cancer cells.
